# Thymic Involution in Viable Motheaten (me^υ^) Mice is Associated with a Loss of Intrathymic Precursor Activity

**DOI:** 10.1155/1992/68954

**Published:** 1992

**Authors:** Sandra M. Hayes, Leonard D. Shultz, Dale L. Greiner

**Affiliations:** 1 Department of Pathology University of Connecticut Health Center Farmington Connecticut 06030 USA; 2 The Jackson Laboratory Bar Harbor Maine 04609 USA; 3 Department of Medicine University of Massachusetts 55 Lake Avenue North Worcester Massachusetts 01655 USA

**Keywords:** Viable motheaten mice, thymocytopoiesis, involution, aging, steroids, thymus

## Abstract

Mice homozygous for the viable motheaten (me^υ^) allele manifest abnormalities in thymocytopoiesis, are severely immunodeficient, and develop autoimmune disorders early in life. Premature thymic involution occurs in me^υ^/me^υ^ mice, and their bone marrow prothymocytes are unable to repopulate the thymus of adoptive recipients following intravenous (i.v.) transfer. However, analysis of thymocytopoiesis following intrathymic (i.t.) adoptive transfer of bone marrow from me^υ^/me^υ^ mice demonstrates the presence of normal numbers of prothymocytes. To investigate intrathymic development in me^υ^/me^υ^ mice, we determined intrathymic precursor cell number and activity. Dual labeling analyses showed that an involuted me^υ^/me^υ^ thymus is relatively enriched (fivefold) in CD4^–^ CD8^–^ thymocytes (intrathymic precursor phenotype) compared with wild-type (+/+) thymus. However, thymocytes from me^υ^/me^υ^ mice were deficient in precursor activity when adoptively transferred i.t. into irradiated recipients. Thymocytes recovered from the involuted thymus of aged or steroid-treated normal mice also displayed reduced precursor activity. However, the phenotypic profile of thymocyte subsets from steroid-treated mice was enriched in single positive cells (mature phenotype) and was distinctly different from the subset distribution of thymocytes in me^υ^/me^υ^ and aged mice. These results suggest that intrathymic precursor activity in me^υ^/me^υ^ mice is decreased, and may be reflective of decreased prothymocyte seeding to the thymus *in vivo*, In addition, the results suggest that the thymic involution in me^υ^/me^υ^ mice is not due solely to effects of corticosteroids.


*Developmental Immunology*, 1992, Vol. 2, pp. 191–205
Reprints available directly from the publisher
Photocopying permitted by license only

© 1992 Harwood Academic Publishers GmbH
Printed in the United Kingdom

### Thymic Involution in Viable Motheaten (*me^v*) Mice is Associated with a Loss of Intrathymic Precursor Activity

SANDRA M. HAYES,† LEONARD D. SHULTZ,*‡ and DALE L. GREINER†§

†*Department of Pathology, University of Connecticut Health Center, Farmington, Connecticut 06030*
‡*The Jackson Laboratory, Bar Harbor, Maine 04609*
§*Department of Medicine, University of Massachusetts, 55 Lake Avenue North, Worcester, Massachusetts 01655*

Mice homozygous for the viable motheaten (*me^v*) allele manifest abnormalities in thymocytopoiesis, are severely immunodeficient, and develop autoimmune disorders early in life. Premature thymic involution occurs in *me^v/me^v* mice, and their bone marrow prothymocytes are unable to repopulate the thymus of adoptive recipients following intravenous (i.v.) transfer. However, analysis of thymocytopoiesis following intrathymic (i.t.) adoptive transfer of bone marrow from *me^v/me^v* mice demonstrates the presence of normal numbers of prothymocytes. To investigate intrathymic development in *me^v/me^v* mice, we determined intrathymic precursor cell number and activity. Dual labeling analyses showed that an involuted *me^v/me^v* thymus is relatively enriched (fivefold) in $CD4^-CD8^-$ thymocytes (intrathymic precursor phenotype) compared with wild-type (+/+) thymus. However, thymocytes from *me^v/me^v* mice were deficient in precursor activity when adoptively transferred i.t. into irradiated recipients. Thymocytes recovered from the involuted thymus of aged or steroid-treated normal mice also displayed reduced precursor activity. However, the phenotypic profile of thymocyte subsets from steroid-treated mice was enriched in single positive cells (mature phenotype) and was distinctly different from the subset distribution of thymocytes in *me^v/me^v* and aged mice. These results suggest that intrathymic precursor activity in *me^v/me^v* mice is decreased, and may be reflective of decreased prothymocyte seeding to the thymus *in vivo*. In addition, the results suggest that the thymic involution in *me^v/me^v* mice is not due solely to effects of corticosteroids.

KEYWORDS: Viable motheaten mice, thymocytopoiesis, involution, aging, steroids, thymus.

### INTRODUCTION

Mice homozygous for the motheaten (*me*) or viable motheaten (*me^v*) alleles display numerous defects in lymphopoiesis. These defects include a paucity of surface immunoglobulin-positive ($Ig^+$) B cells and abnormal peripheral T-cell functions (Green and Shultz, 1975; Shultz et al., 1984; Greiner et al., 1986; Shultz and Sidman, 1987; Shultz, 1988). Furthermore, a number of autoimmune disorders develop that include hypergamma-globulinemia, autoantibody production, and immune complex glomerulonephritis (Kincade, 1981; Shultz and Sidman, 1987; Shultz, 1988). The usual cause of death is a characteristic hemorrhagic macrophagic pneumonitis that is believed to be autoimmune in origin (Shultz and Sidman, 1987; Shultz, 1988). This autoimmune syndrome can be transferred to irradiated recipients using *me^v/me^v* bone marrow stem cells (Kincade, 1981; Shultz, 1988).

Developmental abnormalities are evident in cells that express the enzyme terminal deoxynucleotidyl transferase (TdT; presumptive T- and B-lymphoid stem cells) in *me/me* and *me^v/me^v* bone marrow and thymus (Landreth et al., 1981; Greiner et al., 1986). These bone marrow and thymus $TdT^+$-cell developmental defects appear to be associated with the defects in thymocytopoiesis and may have a causal relationship to the peripheral T- and B-lymphocyte abnormalities. Moreover, at least some of the $TdT^+$- (Landreth et al., 1981; Greiner et al., 1986) and B-cell progenitor cell (McCoy et al., 1985; Greiner et al., 1986; Kincade, 1987) developmental abnormalities,

*Corresponding author. Present address: The Jackson Laboratory, Bar Harbor, Maine 04609.

when analyzed in selective *in vitro* assay systems (Medlock et al., 1987; Hayashi et al., 1988), appear to be secondary to bone marrow microenvironmental defects.

Studies on thymocytopoiesis in *me/me* and *me$^v$/me$^v$* mice have demonstrated a premature thymic involution, commencing as early as 4 weeks of age (Greiner et al., 1986; Shultz and Sidman 1987; Shultz, 1988). Furthermore, we have shown that both *me/me* and *me$^v$/me$^v$* bone marrow prothymocytes fail to repopulate the thymus of irradiated recipients after intravenous (i.v.) transfer, but generate normal numbers of thymocytes following intrathymic (i.t.) adoptive transfer (Greiner et al., 1986). Mixing of *me$^v$/me$^v$* bone marrow with normal bone marrow restores the ability of *me$^v$/me$^v$* prothymocytes to generate thymocytes after i.v. injection (Komschlies et al., 1987), again suggesting that a marrow microenvironmental defect may underlie certain of the early developmental defects observed in lymphopoiesis in *me/me* and *me$^v$/me$^v$* mice.

The apparent prothymocyte homing defect in *me/me* and *me$^v$/me$^v$* bone marrow cells may have *in vivo* consequences, since postnatal maintenance of normal thymocytopoiesis is dependent upon a low, but continual, seeding of the thymus by bone marrow hemopoietic precursors (Scollay and Shortman, 1984; Scollay et al., 1986). Furthermore, the ability of bone marrow precursors to generate thymocytes following entry into the thymus appears to be self-limiting, and will cease within 4 to 5 weeks following thymic entry (Goldschneider et al., 1986; Scollay et al., 1988). Because *me/me* and *me$^v$/me$^v$* bone marrow prothymocytes appear to be unable to repopulate the thymus of irradiated recipients following i.v. transfer, we have postulated that this defect may result in an early loss of intrathymic precursors in *me/me* and *me$^v$/me$^v$* mice, leading to premature thymic involution (Greiner et al., 1986; Komschlies et al., 1987).

We have investigated this hypothesis in the present study using the i.t. adoptive transfer assay to quantitate directly the intrathymic precursor activity prior to and during thymic involution in *me$^v$/me$^v$* mice. Our results indicate that the pool of intrathymic precursors in *me$^v$/me$^v$* mice decreases early in life, in association with the onset of thymic involution. Furthermore, the thymic involution observed in *me$^v$/me$^v$* mice does not appear to be similar to that induced following administration of corticosteroids.

### RESULTS

### Phenotypic Characteristics of *me$^v$/me$^v$* Thymocytes During Thymic Involution

Investigations of murine thymocyte subpopulations have revealed a large diversity of cell subsets (Scollay et al. 1988; Wilson et al., 1988). Four major phenotypic populations have been extensively characterized, and are identified as CD4$^-$CD8$^-$ (double negative, DN), CD4$^+$CD8$^+$ (double positive, DP), and CD4$^+$CD8$^-$ or CD4$^-$CD8$^+$ (single positive, SP). Because the thymus of *me$^v$/me$^v$* mice has been shown to undergo premature thymic involution, commencing as

TABLE 1
Subpopulations of Thymocytes in Various Ages of C57BL/6J-*me$^v$/me$^v$* Mice[a]

| Genotype | Thymus cell number (x10$^{-6}$) | Animal age (weeks) | Thymocyte subpopulations | | | |
|---|---|---|---|---|---|---|
| | | | CD4$^-$CD8$^-$ (%) | CD4$^+$CD8$^+$ (%) | CD4$^+$CD8$^-$ (%) | CD4$^-$CD8$^+$(%) |
| *me$^v$/me$^v$* | 1.6 | 7 | 31.3 | 33.1 | 7.5 | 27.5 |
| | 3.8 | 6 | 15.8 | 48.7 | 15.8 | 19.7 |
| | 9.0 | 4 | 24.4 | 43.2 | 21.8 | 10.8 |
| | 13.0 | 4 | 14.0 | 47.8 | 28.3 | 9.9 |
| | 58.0 | 5 | 5.5 | 69.4 | 15.3 | 15.3 |
| | 72.5 | 4 | 11.6 | 65.0 | 15.7 | 7.6 |
| | 135.0 | 5 | 8.7 | 71.7 | 13.0 | 6.9 |
| | 150.0 | 4 | 4.0 | 88.7 | 5.0 | 2.2 |
| | 270.0 | 4 | 4.1 | 85.1 | 8.1 | 2.8 |
| Wild-type (+/+) | 285.0±37.7 | 4 | 3.9±0.1 | 80.7±1.3 | 9.4±0.4 | 6.0±0.9 |

[a]Thymocytes from various aged C57BL6J-*me$^v$/me$^v$* mice were analyzed for expression of CD4 and CD8 antigens using two-color immunofluorescence staining (see Materials and Methods). Data represent individual *me$^v$/me$^v$* thymuses except for the C57BL/6J+/+ data that represent the mean percentage±standard deviation for three thymuses from 4-week-old mice.

early as 4 weeks of age (Greiner et al., 1986; Shultz and Sidman, 1987; Shultz, 1988), we examined the phenotypic characteristics of the thymocytes prior to and during this involution process. As shown in Table 1 and Figure 1B, the loss of cellularity in the *me$^v$/me$^v$* thymus is evident as early as 4 weeks of age compared with untreated control B6+/+ mice. The loss of cellularity, variable between 4 and 7 weeks of age, is accompanied by a loss in the absolute numbers of DN, DP, and SP thymocytes compared with weanling B6 wild-type mice, with losses of 95%, 99.5%, and 98.4%, respectively, in the most severely involuted thymuses. The relative proportions of each unique thymocyte subset, however, were not as severely altered (Fig. 1B). There was a relative increase in the proportion of DN thymocytes in the most severely involuted thymus, approaching 30% of the total thymocyte population as compared to that of approximately 5% DN cells in the thymus of control B6+/+ mice. In the severely involuted thymuses (less than 13x10$^6$ total thymocytes), the DP and SP populations were approximately equal, each comprising 33–49% of the remaining cells present, whereas in normal B6+/+ mice, the DP and SP populations normally comprise approximately 80% and 15%, respectively, of a thymocyte population (Scollay et al., 1988). However, during the involution process, as thymus cellularity decreased, but prior to the most severely involuted state, the relative proportions of each

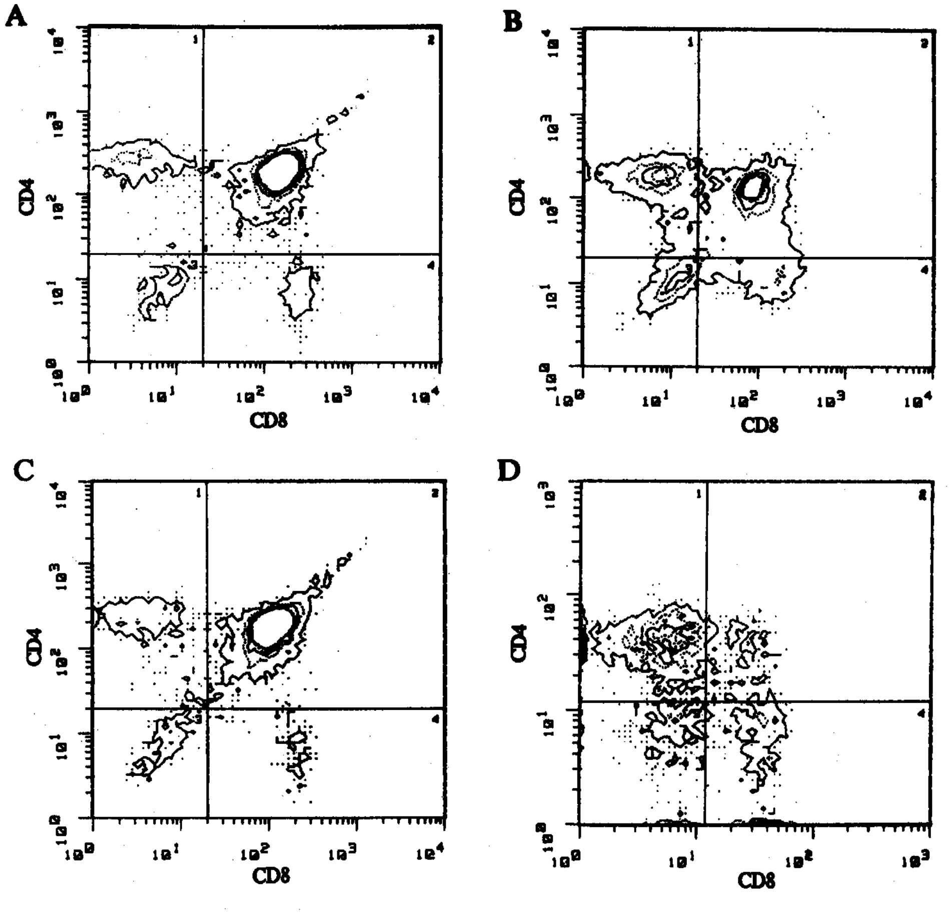

FIGURE 1 Dual labeling analysis of normal and involuted thymuses with anti-CD4 and anti-CD8 monoclonal antibodies. (A) Normal thymus with a cellularity of 250x10$^6$ cells. Percentage of thymocytes in each quadrant: 1 ($CD4^+CD8^-$), 10.5%; 2 ($CD4^+CD8^+$), 81.3%; 3 ($CD4^-CD8^-$), 3.4%; and 4 ($CD4^-CD8^+$), 4.8%. (B) Involuted *me$^v$/me$^v$* thymus with a cellularity of 13x10$^6$ cells. Percentage of thymocytes in each quadrant: 1, 28.3%; 2, 47.8%; 3, 14.0%; and 4, 9.9%. (C) Involuted thymus from an 18-month-old normal B6 wild-type mouse with a cellularity of 42x10$^6$ cells. Percentage of thymocytes in each quadrant: 1, 9.0%; 2, 83.9%; 3, 3.9%; and 4, 3.2%. (D) Involuted thymus with a cellularity of 8x10$^6$ cells from a steroid-treated 4-week-old normal B6 mouse. Percentage of thymocytes in each quadrant: 1, 50.9%; 2, 12.3%; 3, 17.4%; and 4, 19.4%.

of the major thymocyte subsets approximated that observed in normal mice (Table 1).

### Intrathymic Precursor Activity of *me*$^{v}$/*me*$^{v}$ Thymocytes During Thymic Involution

The relative proportion of DN thymocytes was increased in the involuted *me*$^{v}$/*me*$^{v}$ thymus (Table 1 and Figure 1B), although the absolute number of DN thymocytes was decreased (Table 1). The increased percentage of DN thymocytes may be reflective of a compensatory enrichment in intrathymic precursors (i.e., DN thymocytes that possess the ability to repopulate the thymus of irradiated recipients upon adoptive transfer). Alternatively, the DN thymocytes remaining in the involuted thymuses of *me*$^{v}$/*me*$^{v}$ mice may be $CD3^{+}$ DN thymocytes with no precursor activity upon adoptive transfer (Scollay et al., 1988). To investigate these various possibilities, we used the i.t. adoptive transfer assay (Goldschneider et al., 1986; Scollay et al., 1988; Shortman et al., 1988) to quantitate the intrathymic precursor activity of thymocytes in *me*$^{v}$/*me*$^{v}$ mice. This assay system was used because intrathymic precursors poorly repopulate the thymus following i.v. adoptive transfer (Kadish and Basch, 1977; Fowlkes et al., 1985; Scollay et al., 1988), and intrathymic precursor activity is readily quantified using the i.t. adoptive transfer assay (Goldschneider et al., 1986; Scollay et al., 1988).

Thymocytes recovered from *me*$^{v}$/*me*$^{v}$ mice with involuted thymuses were relatively deficient, on a per cell basis, in their ability to repopulate the thymus of adoptive recipients as compared to thymocytes recovered from B6+/+ mice. The i.t. transfer of 1.2 to $1.9\times10^{6}$ thymocytes from *me*$^{v}$/*me*$^{v}$ mice with $2.1\pm1.0\times10^{6}$ cells/thymus resulted in the generation of $<1\times10^{6}$ (nondetectable) ($n$=6) donor-origin cells on day 14, and the transfer of $1\times10^{6}$ or $2.5\times10^{6}$ thymocytes from B6+/+ mice resulted in the generation of $3.9\pm1.0\times10^{6}$ ($n$=4) and $8.0\pm4.3\times10^{6}$ ($n$=3) donor-origin cells, respectively, on day 14. In addition, it was of interest to compare the "total" precursor population in the thymus of *me*$^{v}$/*me*$^{v}$ mice with that of normal B6+/+ mice during the involution process. To compare this directly, the relative intrathymic precursor activity of each thymus, which takes into account the total cellularity of the thymus, hence the total precursor pool within the thymus, was calculated (see Materials and Methods).

Thymocytes from 4-week-old *me*$^{v}$/*me*$^{v}$ mice clustered into two distinct groups when the relative intrathymic precursor activity was calculated (Fig. 2A). In one group, the relative intrathymic precursor activity was equivalent to that of age-matched B6+/− littermates. In the second group, the relative intrathymic precursor activity was decreased in relation to that of B6+/− littermates (Fig. 2A). In 5-, 6-, and 7-week-old *me*$^{v}$/*me*$^{v}$ mice, the relative intrathymic precursor activity was reduced in relation to that of age-matched B6+/− littermates, approaching nondetectable levels of precursor activity in 60% (3/5) of 6-week-old *me*$^{v}$/*me*$^{v}$ mice and 100% (3/3) of 7-week-old *me*$^{v}$/*me*$^{v}$ mice. No differences were observed in the relative intrathymic precursor activity of B6+/− littermates between 4 and 7 weeks of age (Fig. 2A), which, as observed in bone marrow prothymocyte activity (Komschlies et al., 1987), were comparable to that of wild-type B6+/+ mice (data not shown). Furthermore, in 3-week-old *me*$^{v}$/*me*$^{v}$ mice, we observed a decrease in intrathymic precursor activity prior to detectable thymic involution, suggesting that the loss of intrathymic precursor activity precedes thymic involution (data not shown).

Due to the variability in intrathymic precursor activity observed in involuting *me*$^{v}$/*me*$^{v}$ thymuses, especially in 4-week-old *me*$^{v}$/*me*$^{v}$ mice, we determined whether the extent of thymic involution in *me*$^{v}$/*me*$^{v}$ mice (loss in total cellularity) was related to the level of intrathymic precursor activity. As shown in Fig. 2B, there is a strong linear correlation ($r^2$=0.872) between the cellularity of the *me*$^{v}$/*me*$^{v}$ thymus and the relative intrathymic precursor activity detectable during involution; as thymic cellularity decreased, the amount of intrathymic precursor activity correspondingly decreased.

### Phenotypic Characteristics of Thymocytes in Aged B6 Wild-Type Mice During Thymic Involution

Motheaten (*me*/*me*) and *me*$^{v}$/*me*$^{v}$ mice have expected lifespans of 22 and 61 days, respectively (Shultz and Sidman, 1987; Shultz, 1988), and we have previously suggested (Medlock et al., 1986) that these mice may be representative of an accelerated aging process in the immune system. Therefore, it was of interest to compare directly

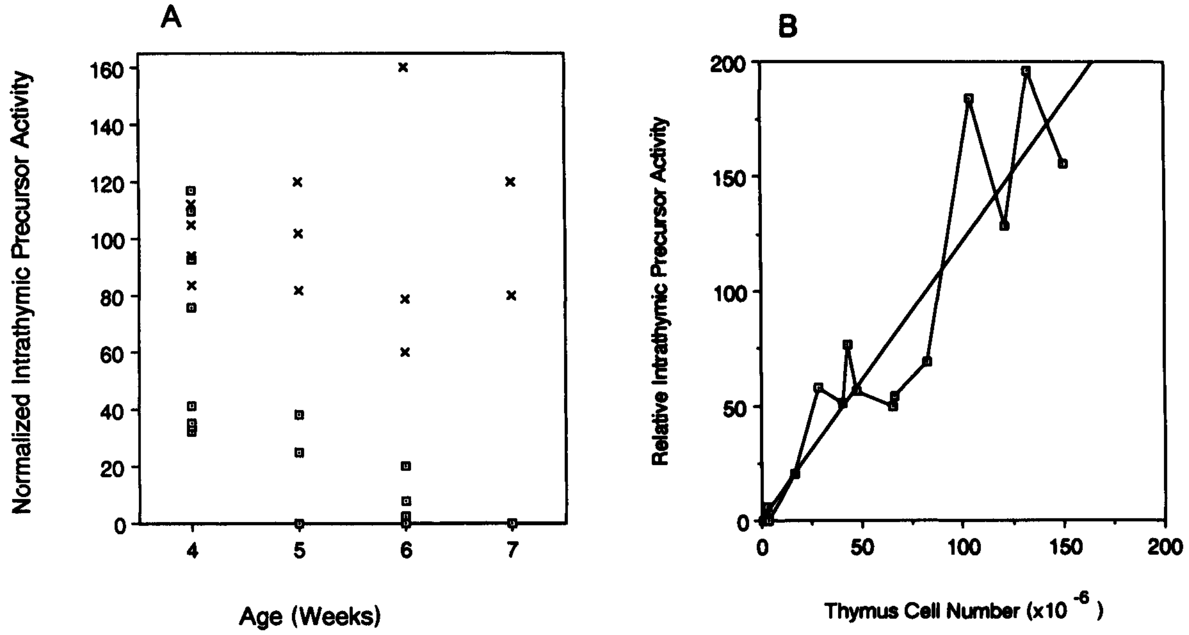

FIGURE 2 Thymocytes from $me^v/me^v$ and age-matched B6+/− littermate mice (Ly 5.2) were tested for precursor activity following intrathymic adoptive transfer into irradiated B6-Ly5.1 congenic recipients. Relative intrathymic precursor activity was calculated as described (see Materials and Methods). Values represent data from individual thymuses. (A) Relative intrathymic precursor activity normalized to that of control B6+/− littermates as a function of age. Open squares represent $me^v/me^v$ thymuses and x's represent age-matched B6+/− littermate thymuses. (B) Relative intrathymic precursor activity as a function of thymus cellularity ($r^2$=0.872).

the thymic involution of $me^v/me^v$ mice with that observed in normal mice during aging.

As observed in an $me^v/me^v$ involuting thymus, there is a significant decrease in the absolute number of each of the four major thymocyte subsets (DN, 76%; DP, 92%; SP, 80%) in the severely involuted thymuses from aged normal mice as compared to thymocyte subsets present in weanling (4–6-week-old) B6+/+ mice (Table 2). However, the relative proportion of each of the major thymocyte subsets was consistent in aging B6+/+ mice through 14–15 months of age, although the cellularity of the older 14–16-month thymuses was decreased 60 to 90% with respect to that of weaning B6+/+ mice (Table 2). The staining profile of a typical older thymus (cellularity of 42x $10^6$) is shown in Fig. 1C.

### Intrathymic Precursor Activity of Thymocytes in Aged Mice During Thymic Involution

Thymocytes from weanling (1-month-old) B6+/+ mice were comparable to that of weanling B6+/− littermate mice in intrathymic precursor activity (Figs. 2A and 3A). By 11 to 13 months of age, however, the intrathymic precursor activity of B6+/+ mice was decreased by 57 to 87% as compared to that of 1-month-old B6+/+ mice and continued to decrease with time to essentially nondetectable levels (98% decrease) of intrathymic precursor activity in 100% (4/4) of 16–17-month-old mice (Fig. 3A). As observed in involuting $me^v/me^v$ thymuses, the decrease in thymus cellularity exhibited a strong linear correlation ($r^2$=0.900) with the loss of intrathymic precursor activity with age (Fig. 3B).

### Prothymocyte Activity in the Bone Marrow of Aged Mice

Viable motheaten ($me^v/me^v$) mice with severely involuted thymuses still contain normal numbers of bone marrow prothymocytes as quantitiated by the i.t. adoptive transfer assay system, but no detectable intrathymic precursor activity (Greiner et al., 1986; Komschlies et al., 1987; Fig. 2). Therefore, we determined whether the loss of intrathymic precursor activity in B6+/+ aged

TABLE 2
Effect of Age on Subpopulations of Thymocytes in C57BL/6J+/+ Mice[a]

| Thymus cell number ($\times10^{-6}$) | Animal age (months) | Thymocyte subpopulations | | | |
|---|---|---|---|---|---|
| | | $CD4^-CD8^-$ (%) | $CD4^+CD8^+$ (%) | $CD4^+CD8^-$ (%) | $CD4^-CD8^+$ (%) |
| 18 | 15 | 4.8[b] | 71.7 | 15.1 | 5.8 |
| 20 | 14 | 3.6 | 76.0 | 15.1 | 3.8 |
| 23 | 18 | 11.6 | 43.6 | 19.6 | 12.5 |
| 42 | 18 | 1.8 | 84.3 | 8.8 | 3.6 |
| 43 | 12 | 2.5 | 87.2 | 8.0 | 2.1 |
| 53 | 18 | 4.7 | 77.1 | 13.5 | 4.7 |
| 60 | 3 | 3.1 | 81.3 | 10.9 | 3.6 |
| 60 | 14 | 4.8 | 73.4 | 14.3 | 5.8 |
| 67 | 12 | 3.3 | 86.3 | 7.3 | 3.1 |
| 72 | 15 | 4.0 | 77.4 | 12.8 | 4.9 |
| 77 | 8 | 3.9 | 76.9 | 14.1 | 4.2 |
| 108 | 8 | 4.1 | 78.2 | 12.5 | 4.1 |
| 110 | 12 | 3.9 | 83.9 | 9.0 | 3.3 |
| 175 | 3 | 3.3 | 82.9 | 10.0 | 3.6 |
| 181.7±10.4 | 1 | 3.2±0.7 | 83.2±3.0 | 9.0±1.4 | 4.5±1.2 |

[a]Thymocytes from various aged C57BL/6J+/+ mice were analyzed for expression of CD4 and CD8 antigens using two-color immunofluorescence staining (see Materials and Methods). Data represent individual thymuses except for the 1-month-old group that represents the mean percentage±standard deviation of three individual thymuses.
[b]The mean percentage of the $CD4^-CD8^-$ subset excludes $sIg^+$ cells in the thymus.

mice is associated with a decrease in bone marrow prothymocyte activity. Bone marrow was recovered from 12-month-old B6+/+ mice (57 to 87% decrease in intrathymic precursor activity) and injected both i.v. and i.t. into young (4–6-week-old) irradiated adoptive recipients to determine their relative bone marrow prothymocyte activity. As shown in Table 3, in contrast to that observed in $me^v/me^v$ mice and as shown previously by other investigators (Hirokawa et al., 1986), the bone marrow of aged mice is able to repopulate the thymus of young adoptive recipients following i.v., as well as i.t., adoptive transfer. Furthermore, on a per cell basis, bone marrow from 1-year-old donors does not differ significantly from that of weanling (1-month-old) bone marrow in relative prothymocyte activity. For the i.v. adoptive transfer system, the relative prothymocyte activity is 219±109 (mean±S.D.) units for young donors and 421±147 units for aged ones. Similar results are observed for the i.t. adoptive transfer system in which the relative prothymocyte activity is 10,410±7,563 units for young bone marrow and 16,245±8,189 units for aged marrow.

To investigate further the possible mechanisms that account for thymic involution in aged mice, especially since bone marrow precursor activity in older mice is comparable to that of young mice (Table 3), we determined the ability of young and older (12-month-old) bone marrow to repopulate the thymuses of older (10-month-old) irradiated recipients. Surprisingly, there were no significant differences ($p>0.1$ for the i.v. assay; $p>0.5$ for the i.t. assay) in the number of thymocytes generated by either young or old marrow on a per cell basis in older recipients as compared to that of young recipients. The relative prothymocyte activities calculated for both adoptive transfer assays are 477±127 units (i.v.) and 15,912±3,917 units (i.t.) for young bone marrow and 372±58 units (i.v.) and 14,070±9,478 units (i.t.) for aged marrow.

### Phenotypic Characteristics of Thymocytes in Corticosteroid-Treated Mice Following Thymic Involution

To investigate whether the thymic involution observed in $me^v/me^v$ mice resembled that of corticosteroid-induced thymic involution, normal B6+/+ mice were injected with dexamethasone (0.5 mg), and the thymus analyzed 48 h later. Steroid treatment resulted in a 97% decrease in cellularity, and a decrease in the absolute numbers of each of the four major thymocyte subsets (Table 4). The proportions of each of the thymus subsets that were present, however, were different (Fig. 1D) from that observed in an $me^v/me^v$ involuted thymus (Fig. 1B). The proportion of DN thymocytes increased to 19.3±3.1%, similar to that of $me^v/me^v$ thymocytes (range 5.5–32%), but the percentage of DP

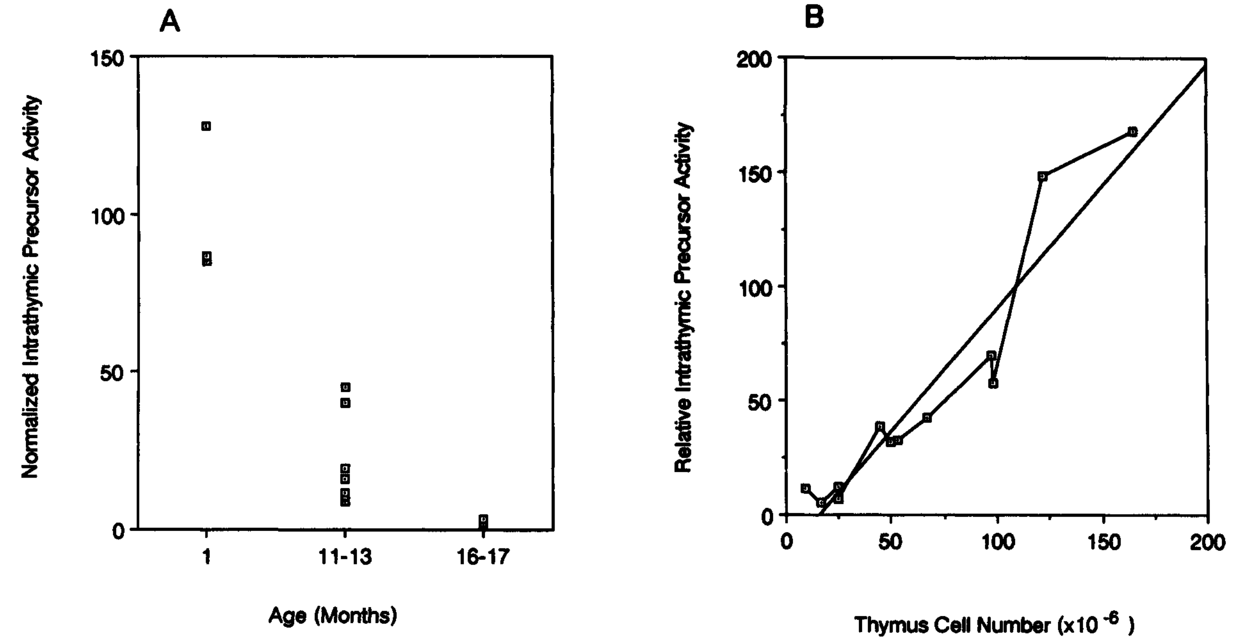

FIGURE 3 Thymocytes from 1-, 11–13-, and 16–17-month-old normal B6+/+ mice (Ly5.2) were tested for precursor activity following intrathymic adoptive transfer into irradiated B6-Ly5.1 congenic recipients. Relative intrathymic precursor activity was calculated as described (see Materials and Methods). Values represent data from individual thymuses. (A) Relative intrathymic precursor activity as a function of age normalized to that of control 4-week-old B6+/+ mice. (B) Relative intrathymic precursor activity as a function of thymus cellularity ($r^2$=0.900).

TABLE 3
Effect of Age on Bone Marrow Prothymocyte Activity in C57BL/6J+/+ Mice[a]

| Age of donor (months) | Age of recipient of (months) | Route injection | Donor-origin thymocytes | |
|---|---|---|---|---|
| | | | Percentage | Cells/thymus $(\times 10^{-6})$ |
| 1 | 1 | i.t. | 67.8±18.7 | 90.5±65.8 |
| 12 | 1 | i.t. | 85.2±11.0 | 81.2±40.9 |
| 1 | 10 | i.t. | 86.4±4.6 | 146.3±36.0 |
| 12 | 10 | i.t. | 78.4±12.3 | 112.6±75.8 |
| 1 | 1 | i.v. | 73.2±10.6 | 95.5±47.4 |
| 12 | 1 | i.v. | 82.1±5.2 | 105.3±36.9 |
| 1 | 10 | i.v. | 94.7±0.9 | 216.3±54.1 |
| 12 | 10 | i.v. | 89.5±6.2 | 148.9±23.2 |

[a]Bone marrow cells from young (1-month-old) and aged (12-month-old) mice were injected i.t. (0.2×10$^6$) or i.v. (10×10$^6$) into irradiated young (1-month-old) or aged (10-month-old) B6+/+ recipients. Twenty days post injection, thymuses were recovered and analyzed for donor-origin thymocytes using the indirect immunofluorescence staining technique (see Materials and Methods). Data represent mean±standard deviation for three or more animals per group.

thymocytes (10.4±3.4%) was decreased relative to involuted *me*$^v$/*me*$^v$ thymocytes (range 33–72%), and SP thymocytes were the predominant thymocyte subset (65.8±5.4%) in steroid-treated mice (Table 4 and Fig. 1D).

### Intrathymic Precursor Activity of Thymocytes in Steroid-Treated Mice

Thymocytes recovered from steroid-treated B6+/+ mice were tested for their intrathymic precursor activity in the i.t. adoptive transfer assay. The thymocyte repopulating activity of steroid-treated thymocytes (1.1±0.1 units) was decreased more than 99% when compared to the relative thymocyte repopulating activity of age-matched untreated B6+/+ (392.2±185.8 units) mice.

### Prothymocyte Activity in the Bone Marrow of Steroid-Treated Mice

The high sensitivity of intrathymic precursors to steriods was surprising in light of previously published results detailing the relative steroid resistance of bone marrow prothymocytes (Greiner et al., 1982). To confirm this result, bone marrow cells were recovered from the same steriod-treated mice that were used as donors of thymocytes (see Table 4), and were injected both i.t. and i.v. into adoptive recipients. As expected, on a per cell basis and as previously reported

TABLE 4
Dexamethasone Sensitivity of Thymocyte Subpopulations in C57BL/6J+/+ Mice[a]

| Dexamethasone treatment | Thymus cell number ($\times 10^{-6}$) | Thymocyte subpopulations[b] | | | |
|---|---|---|---|---|---|
| | | $CD4^-CD8^-$ (%) | $CD4^+CD8^+$ (%) | $CD4^+CD8^-$ (%) | $CD4^-CD8^+$ (%) |
| + | 7.5±4.1 | 19.3±3.1[b] | 10.4±3.4 | 48.1±4.6 | 17.8±3.8 |
| – | 173.3±10.4 | 3.0±0.6 | 82.4±3.9 | 10.1±2.1 | 4.5±1.2 |

[a]Four-week-old C57BL/6+/+ mice were injected with dexamethasone (0.5 mg) and the thymuses were recovered for analysis 48 h later. Thymocytes from these mice were analyzed for expression of CD4 and CD8 antigens using two-color immunofluorescence staining (see Materials and Methods). Data represent the mean percentage±standard deviation for four mice in the dexamethasone-treated group and for three mice in the untreated group.
[b]The mean percentage for $CD4^-CD8^-$ thymocytes excludes surface $Ig^+$ cells in the thymus.

(Greiner et al., 1982), the number of thymocytes generated by equivalent doses of untreated and steroid-treated bone marrow was essentially equal (Table 5). However, because the cellularity of the steroid-treated bone marrow was reduced approximately 60%, the total number of prothymocytes in steroid-treated marrow was approximately 40% of that of normal marrow. Although variable, when the same bone marrow was analyzed by the i.t. assay, similar results were observed. By using a standard cell dose of $2.0\times10^5$ cells/recipient, the total prothymocyte activity per leg (femur plus tibia) detectable in the marrow of steroid-treated mice was approximately 30% of that of untreated mice (Table 5).

### Expression of CD3 and Surface Immunoglobulin in the DN Population

The DN thymocyte population is the only thymocyte subset demonstrated to be able to repopulate the thymus of adoptive recipients in both the i.v. and i.t. adoptive transfer systems (Scollay et al., 1988). However, $CD4^-CD8^-$ thymocytes that express CD3 do not generate thymocytes upon i.t. injection into adoptive recipients (Crispe et al., 1987; Scollay et al., 1988; Shortman et al., 1988). In addition, approximately 1% of cells in the normal thymus are surface $Ig^+$ B lymphocytes (Andreu-Sanchez et al., 1990). Surface $Ig^+$ cells could be contaminants in the DN ($CD4^-CD8^-$) population, and would also fail to generate thymocytes upon i.t. adoptive transfer into irradiated recipients. Therefore, DN thymocytes that expressed either CD3 or sIg were quantitated in the thymus of normal B6+/+ 4–6-week mice as well as in involuted thymuses (Table 6). Even though the percentage of DN thymocytes is greater in the severely involuted thymuses (<25× $10^6$ thymocytes), the proportion of $CD3^+$ or $sIg^+$ DN cells approximates that observed in normal thymuses (approximately 50%).

### DISCUSSION

In the present study, we have demonstrated that $me^v/me^v$ involuted thymuses display a loss in all four of their major phenotypic thymocyte subsets (DN, DP, SP) by 4 to 7 weeks of age. Prior to this loss of thymocytes, a decrease in the intrathymic precursor activity was observed. This decrease was evident as early as 3 weeks of age in $me^v/me^v$ mice whose thymuses have not lost cellularity. At 4 weeks of age, approximately half of the $me^v/me^v$ mice examined had reduced intrathymic precursor activity, and the majority of the animals studied at 6 to 7 weeks of age demonstrated reduced activity. Although a relative increase in the percentage of DN thymocytes was observed

TABLE 5
Dexamethasone Sensitivity of Bone Marrow Prothymocytes in C57BL/6J+/+ Mice[a]

| Route of injection | Dexamethasone treatment | Number of donor-origin thymocytes ($\times10^6$) | Relative bone marrow prothymocyte activity/femur+tibia ($\times10^{-6}$)[b] |
|---|---|---|---|
| i.v. | – | 52.7±22.6 | 178 |
| i.v. | + | 45.4±21.1 | 66 |
| i.t. | – | 111.8±78.6 | 18,894 |
| i.t. | + | 80.4±42.3 | 5,869 |

[a]Four-week-old C57BL/6J+/+ mice were injected with dexamethasone (0.5 mg) and the thymuses were recovered 48 h later. Bone marrow cells from dexamethasone and untreated mice were injected i.t. ($0.2\times10^6$) or i.v. ($10\times10^6$) into irradiated B6 Ly 5 congenic recipients. Twenty days post injection, thymuses were recovered and the percentage of donor-origin thymocytes was determined by indirect immunofluorescence staining (see Materials and Methods). Data represent the mean±standard deviation for three or more mice per group.
[b]Relative bone marrow prothymocyte activity was calculated as follows: number of donor-origin thymocytes divided by the number of cells injected times the total number of donor bone marrow cells per leg (femur plus tibia) (see Materials and Methods).

in the involuting thymus of $me^v/me^v$ mice, there was a decrease in the absolute number of DN cells relative to that of normal age-matched B6+/+ mice. Because only DN thymocytes are able to generate the other three phenotypic thymocyte subsets (DP and SP) *in vivo* in adoptive recipients (Scollay et al., 1988; Shortman et al., 1990), our observations would suggest that the intrathymic precursors, although reduced in absolute numbers, may be relatively enriched in involuting $me^v/me^v$ thymuses. However, the DN thymocytes that were present in the severely involuted $me^v/me^v$ thymuses were deficient in their ability to generate thymocytes upon transfer to irradiated adoptive recipients.

It has been calculated that as few as $1\times10^3$ purified DN thymocytes can generate detectable levels of donor-origin thymocytes in an irradiated recipient's thymus, while up to $6\times10^5$ DN thymocytes ($2\times10^7$ unfractionated thymocytes) are required for saturation repopulation of the thymus of an irradiated recipient (Scollay et al., 1988). We injected in our experiments at least $5\times10^5$ thymocytes from each involuted thymus into the irradiated recipient's thymus. Because, in the severely involuted thymus, at least 20% of the thymocytes were DN (Table 1), at least 100,000 DN cells were injected into the thymus of each of the adoptive recipients. Our failure to find evidence of intrathymic precursor activity following injection of approximately 100-fold more DN thymocytes than should have been necessary to generate thymocytes in adoptive recipients (and approximately fourfold more DN thymocytes on a per cell basis than that present in normal thymus that gave high levels of repopulation in adoptive recipients) suggests that at a maximum, less than 1% of the DN thymocytes in the severely involuted $me^v/me^v$ thymuses could have had precursor activity.

The DN thymocytes that are present in normal mice, however, are composed of at least 11 different subsets, each with different functional capabilities and kinetics of thymocyte repopulation in adoptive recipients (Scollay et al., 1988). Also contained within these various thymocyte subsets are DN thymocytes that express either CD3 or surface Ig and are unable to function as intrathymic precursors in adoptive transfer assay systems (Scollay et al., 1988). In addition, variations in the DN $CD3^+$ population with respect to mouse strains and to the class of T-cell receptor expressed occurs (Shortman et al., 1990). Consequently, we analyzed the DN thymocyte population in the involuted thymuses to determine whether there was an increase in the percentage of DN cells expressing CD3 using the 2C11 monoclonal antibody that detects a portion of the CD3 complex expressed by both alpha/beta- and gamma/delta-expressing T cells. An increase in

TABLE 6
Subpopulations of Double Negative ($CD4^-CD8^-$) Thymocytes in Involuted Thymuses[a]

| Animal age (months) | Thymus cell number ($\times10^{-6}$) | $CD4^-CD8^-$ thymocytes (%) | Percentage of $CD4^-CD8^-$ thymocytes that are: | |
|---|---|---|---|---|
| | | | $CD3^+$ or $sIg^+$ | $CD3^-$ $sIg^-$ |
| Untreated (1 month) | 173.3±10.4 | 3.9±0.5 | 51 | 49 |
| Untreated (12 months) | 73.3±33.9 | 4.3±0.5 | 51 | 49 |
| Untreated (18 months) | 53.0[b] | 5.2 | 67 | 33 |
| | 42.0 | 3.5 | 69 | 31 |
| | 23.0 | 24.3 | 81 | 19 |
| Steroid-treated (1 month) | 7.5±4.1 | 23.8±2.2 | 41 | 59 |
| $me^v/me^v$ (1 month) | 270.0[b] | 4.1 | 30 | 70 |
| | 150.0 | 4.5 | 20 | 80 |
| | 13.0 | 14.0 | 49 | 51 |

[a]Thymocytes from normal and involuted thymuses were analyzed for expression of CD4, CD8, CD3, and surface Ig using two-color immunofluorescence staining (see Materials and Methods). Data, unless otherwise marked, represent mean±standard deviations of three or more thymuses.
[b]Due to variability among a single group, data are given for individual thymuses.

the proportion of DN CD3-expressing cells would indicate an increase in the percentage of $CD4^-CD8^-$ thymocytes that would be unable to generate thymocytes in adoptive recipients. We have demonstrated that the involution process in the $me^v/me^v$ mouse does not affect the relative proportion of these cells in the DN subset (Table 6).

In light of our findings, it appears that the thymic involution in $me^v/me^v$ mice may in part be due to the $CD4^-CD8^-CD3^-sIg^-$ subset being unable to generate thymocytes, as demonstrated by their transfer to adoptive recipients. To determine whether these precursors are arrested in development, it will be of interest in future studies to examine additional time points following adoptive transfer of the donor thymocytes to confirm the absence in involuted $me^v/me^v$ thymuses of each of the developmentally distinct populations that have been described. However, based on our inability to demonstrate any intrathymic precursor activity at day 14 following transfer to irradiated recipients, and because of the large excess of DN cells injected, it seems highly unlikely that we have missed a significant population of DN thymocytes in involuting $me^v/me^v$ thymus that possesses precursor activity.

Alternatively, we have previously suggested (Komschlies et al., 1987) that the bone marrow of $me^v/me^v$ mice is deficient in an accessory cell (or factor) that permits repopulation of the thymus of irradiated recipients following i.v. adoptive transfer. Addition of normal bone marrow to $me^v/me^v$ bone marrow restores its i.v. thymus repopulating capacity. Viable motheaten ($me^v/me^v$) bone marrow (as are normal thymocytes), however, is able to repopulate the thymus of irradiated recipients following i.t. injection without additional accessory cell (or factor) requirements. It has been shown previously that a normal thymus contains a population of bone marrow derived "accessory" dendritic cells of myeloid origin (Fowlkes and Pardoll, 1989; Robey et al., 1990; Spangrude and Scollay, 1990; von Boehmer, 1990) that are important in the clonal selection, survival, and differentiation of thymocytes (Robey et al., 1990; von Boehmer, 1990). Codevelopment of such myeloid stem cells following bone marrow i.t. injection appears to be requisite for the subsequent development of intrathymic precursors and donor-origin thymocytes (Spangrude and Scollay, 1990). Because numerous defects in myeloid cells have been described in $me^v/me^v$ mice *in vivo* and *in vitro* (McCoy et al., 1982, 1984; Medlock et al., 1987; Hayashi et al., 1988; Shultz, 1988; Van Zant and Shultz, 1989), the lack of these bone marrow derived dendritic cells in an $me^v/me^v$ thymus may result in the absence, death, and/or functional inactivity of $me^v/me^v$ intrathymic precursors. However, cell mixture experiments of $me^v/me^v$ thymocytes with normal bone marrow cells failed to "rescue" or provide evidence for suppression of the thymus repopulating capacity of $me^v/me^v$ DN thymocytes following i.t. adoptive transfer into irradiated recipients (unpublished observations). These results suggest that $me^v/me^v$ DN thymocytes, if they in fact are intrathymic precursors, may be anergic to such regulatory signals. It does not rule out, however, that these positive signals may be required only at specific, perhaps early, stages of precursor development. The setup of our assay system, in which "accessory" cells are not added until thymic involution (hence loss of intrathymic precursor activity) has already occurred, may be too late to rescue the intrathymic precursor population.

It was also of interest in these studies to compare the phenotypic and functional characteristics of thymocytes in an involuting $me^v/me^v$ thymus with other models of thymic involution in an attempt to gain insight into the possible mechanisms involved. Thymic involution has been observed in pregnancy, infection, surgery, drugs (including cyclosporin A), steroids, malnutrition, malignancy, and aging (Clarke and Kendall, 1989). Of special interest in our studies was the involution in $me^v/me^v$ thymus with that of thymuses in aged mice, because we have suggested previously (Medlock et al., 1986) that the $me^v/me^v$ mouse may represent an accelerated model of aging in the lymphopoietic system. Thymic involution in normal mice begins soon after puberty, while the animal is still increasing in body weight (Santisteban, 1960). There is a decrease in thymic weight with age, a loss of cortical thymocytes, an increase of thymic adipose tissue, and a general decrease in thymic function. In addition, the thymic atrophy is reflected in the peripheral tissues by a decrease in T-cell functional responses, and an increase in a $CD4^+$ $Pgp\text{-}1^+$ population reflective of memory T cells (Nagelkerken et al., 1991).

As predicted, by 12 to 18 months of age, the thymus cellularity in B6+/+ mice is reduced by approximately 90% in comparison to that of young (4-week-old) B6 mice. Our phenotypic studies have demonstrated that in an involuting aged thymus, essentially normal ratios of the four phenotypic subsets of thymocytes are present. Therefore, in the *in vivo* studies, equivalent numbers of young and aged DN thymocytes were injected into the recipient thymus. Even though this is the case, the 17-month-old thymus is severely deficient in intrathymic precursor activity, and displays essentially no ability to repopulate the thymus of irradiated recipients at the cell doses examined (3–5×$10^6$ cells injected). One 18-month-old thymus, however, did exhibit a variation in the proportions of the major thymocyte subsets (24.3% DN, 43.6% DP, 32.1% SP). When the DN thymocyte population was also stained for CD3 or sIg, it was observed that in this involuted thymus, 80% of the $CD4^-CD8^-$ thymocytes expressed either CD3 or Ig.

It appears, however, that the loss of intrathymic precursor activity with age in mice is not due to a defect in the bone marrow prothymocyte population. We found that on a per cell basis, bone marrow from older mice is as efficient as young marrow in thymus repopulating capacity. Contradictory results exist in regards to the study of the aging process and the immune system. CFU-s activity and basal hemopoietic activity do not appear to be affected by age (Tyan, 1982; Williams et al., 1986). However, other investigators find prothymocyte and thymic activity to be impaired in aged mice (Kay, 1984; Hirokawa et al., 1986). We observed the opposite; the defect is not readily apparent in the thymus, as older irradiated recipients readily supported the generation of donor-origin thymocytes from both young and older marrow as well as did younger irradiated recipients. It must be cautioned that the irradiation model of repopulation may not directly represent the *in vivo* situation in regard to thymus function. Thus, irradiation may induce or reactivate "accessory cells" or factors that promote thymocyte regeneration that are quiescent in intact aged hosts. For example, an irradiated thymus may be induced to secrete increased levels of chemotactic factors (Harr et al., 1989) such as thymotaxin (Imhof et al., 1988; Deugnier et al., 1989). However, it is most likely not thymotaxin, because this compound has been found to be beta-2 microglobulin (Dargemont et al., 1989), and beta-2 microglobulin-deficient mutants have normal development of the thymus (Koller et al., 1990; Zijlstra et al., 1990). Alternatively other, as yet undescribed, factors may have a role in the irradiation induced ability of thymus in aged mice to support thymocyte production in the adoptive transfer system.

Similarly, we have provided evidence that the thymic involution in $me^v/me^v$ mice is not due to increased levels of steroids. Although essentially all intrathymic precursor activity is eliminated by a single dose of dexamethasone, we observed significant differences in the phenotypic characteristics of the remaining thymocyte populations. Thus, in thymuses from steroid-treated mice, a large proportion of the remaining lymphoid cells were SP, and in thymuses from either $me^v/me^v$ or aged mice, a much lower proportion of the lymphoid cells were SP. The characteristics of the thymocyte populations that were present following steroid treatment were similar to those previously reported by numerous investigators (Reichert et al., 1986; Vliet et al., 1986). Of interest in this study was the unexpected finding of the higher steroid sensitivity of intrathymic precursors compared to bone marrow prothymocytes. In the bone marrow, the prothymocyte population is relatively resistant to steroids (Greiner et al., 1982). We confirmed these findings, and we were able to calculate that approximately 60% of bone marrow prothymocyte activity is lost following steroid treatment. This is in marked contrast to the loss of intrathymic precursors observed in the steroid-treated mice, in which greater than 99% of the precursor activity was lost. The loss of intrathymic precursor activity, however, is supported by phenotyping data. Approximately 40% of steroid-resistant DN thymocytes express CD3 or Ig and would be unable to generate thymocytes in adoptive recipients (Table 6). Furthermore, other investigators have shown that steroid treatment depletes $CD5^{lo}$ thymocytes (Scollay and Shortman, 1985), a population that contains intrathymic precursor activity (Fowlkes et al., 1985). Thus, although steroids resulting from the stress induced by the clinical symptoms of the autoimmune disease process may contribute to the early thymic involution observed in $me^v/me^v$ mice, such contribution is most likely a small and insignificant part of the involution process.

### MATERIALS AND METHODS

### Animals

Inbred C57BL/6J (Ly 5.2) and Ly 5 congenic C57BL/6J-Ly 5.1 mice (hereafter referred to as B6 Ly5.2 and B6 Ly5.1, respectively) were purchased from Charles River (Boston, MA) and Harlan Sprague Dawley (Frederick, MD), respectively. C57BL/6J-$me^v/me^v$ mice (hereafter referred to as $me^v/me^v$ mice; Ly 5.2) were obtained from the colony maintained at the Jackson Laboratory by LDS. The inbred C57BL/6 mice are designated as Ly 5.2; the congenic is designated by Ly 5.1 (SJL/J phenotype). This nomenclature is consistent with that recently recommended by Morse et al. (1987), and is opposite from the designation of the monoclonal antibodies commercially available from Dupont/New England Nuclear. Unless otherwise noted, animals were placed into experiments when they were 4–6 weeks of age, and were maintained on commercial chow and acidified chlorinated water *ad libitum*.

### Antibodies

The unconjugated monoclonal antibodies directed against the Ly 5.1 and Ly 5.2 alloantigens were obtained from Dupont/New England Nuclear (Boston, MA) and were developed for immunofluorescence analysis using an $F(ab')_2$ fragment of FITC-conjugated goat antimouse IgG (heavy- and light-chain-specific) obtained from Organon Teknika-Cappel (Malvern, PA). Monoclonal antibodies for use in the dual labeling studies, FITC-conjugated antimouse Lyt-2 and Phycoerythrin (PE)-conjugated antimouse L3T4, were obtained from Becton Dickinson Immunocytometry Systems, Mountain View, CA.

### Dexamethasone-Resistant Thymocytes and Bone Marrow Cells

Dexamethasone-resistant thymocytes and bone marrow cells were obtained 48 h after i.p. injection of 0.5 mg of dexamethasone sodium phosphate (LyphoMed, Inc., Melrose Park, IL) into 4–6-week-old mice (Vliet et al., 1986; Compton et al., 1987).

### Cell Suspensions

Bone marrow cells were obtained by flushing tibias and femurs with cold medium (Hepes buffered RPMI 1640). After repeated gentle pipetting to disperse the cells, the marrow was centrifuged at 150×*g* for 5 min and resuspended in cold medium for cell counting. Thymus cell suspensions were prepared by gently pressing the tissues through a 50-mesh cell sieve followed by washing in cold medium. Cell viability was determined by exclusion of 0.1% trypan blue and was greater than 95% in all cases (Greiner et al., 1986).

### Immunofluorescence Analysis

Thymocytes were labeled for immunofluorescence analysis as previously described (Goldschneider, 1986). Briefly, a single-cell suspension of thymocytes ($1\times10^6$/well) was added to individual wells of 96-well U-bottom microtiter plate and spun at 150×*g* for 2 min. The supernatant was removed, the cells resuspended directly in 10 $\mu$l of a saturating concentration of the primary antibody (anti-Ly 5.1 or anti-Ly 5.2) or medium (control) and incubated for 20 min at 4 °C. The cells were spun at 150×*g* for 2 min, the supernatant removed and the cells were washed twice in cold medium containing 0.13% sodium azide. After the last wash, the cells were resuspended directly in 10 $\mu$l of an appropriate dilution of the developing antibody and incubated for 20 min at 4 °C. After the second incubation, the cells were washed, fixed in buffered formalin, and analyzed by flow microfluorimetry for relative immunofluorescence on a FACS 4 (Becton-Dickinson Immunocytometry System, Sunnyvale, CA). All viable nucleated cells were gated for analysis, and dead cells and red blood cells were excluded electronically. In two-color analysis, thymocytes were incubated with FITC-anti-Lyt 2 alone, with PE-anti-L3T4 alone, or simultaneously with both. In some cases, to exclude the possible contribution of surface $Ig^+$ B cells in the involuted thymuses in the calculation of double negative ($CD4^-CD8^-$) thymocytes, anti-CD4, anti-CD8, and FITC-conjugated goat antimouse IgG were used to stain the thymocytes.

### Irradiation

Recipients received the indicated level of whole body irradiation from a $^{137}Cs$ source (gamma cell 40 irradiator, Atomic Energy of Canada Limited,

Ottawa, Canada) at a dose rate of approximately 95 rads/min 2–6 h prior to bone marrow cell transfer (Greiner et al., 1986).

### Intrathymic Adoptive Transfer Assay System

Details of the intrathymic (i.t.) adoptive transfer assay system for prothymocytes have been described previously (Goldschneider et al., 1986; Greiner et al., 1986; Komschlies et al., 1987; Scollay et al., 1988). Briefly, irradiated (700 rad) recipients received varying numbers of thymocytes or bone marrow cells in 20 μl of media distributed equally between each thymus lobe (10 μl/lobe). Thirteen to fifteen days after thymocyte transfer or 18 to 20 days after bone marrow cell transfer, the thymus was removed, and the percentage and number of donor- and host-origin cells were determined by immunofluorescence analysis of Ly 5.1 (host) and Ly 5.2 (donor) bearing thymocytes using flow microfluorimetry.

### Intravenous Adoptive Transfer Assay System

This assay has been described in detail (Goldschneider et al., 1986; Greiner et al., 1986; Komschlies et al., 1987). Briefly, irradiated recipients (700 rad) were injected i.v. in the tail vein with varying numbers of bone marrow cells (see Results). Twenty to twenty-three days later, the percentage and number of donor- (Ly $5.2^+$) and host- (Ly $5.1^+$) origin thymocytes were determined by immunofluorescence analysis using flow microfluorimetry.

### Calculation of Relative Intrathymic Precursor or Bone Marrow Prothymocyte Activity

To calculate the relative intrathymic or prothymocyte precursor activity of a donor thymus or bone marrow when using either the i.t. or i.v. adoptive transfer assay system, the following formula was used:

$$\frac{\text{Number of thymocytes generated from donor-origin cells}}{\text{Cell dose}} \times \text{donor cell number} = \text{precursor activity}$$

For example, if $1\times10^6$ thymocytes injected from a donor thymus that had $30\times10^6$ cells generated 15 $\times10^6$ donor-origin thymocytes in the adoptive recipient, the total intrathymic precursor activity of the donor thymus would be $450\times10^6$.

### Calculation of Normalized Intrathymic Precursor Activity

Once relative intrathymic precursor activity was calculated for individual thymuses, the mean (±standard deviation) intrathymic precursor activity was calculated for the control thymuses. The experimental thymus intrathymic precursor activity was then normalized as a ratio of the mean control thymus value.

### ACKNOWLEDGMENTS

This study was supported by NIH grant CA20408 and by grant 515 from the American Cancer Society.

*(Received August 11, 1991)*

*(Accepted September 29, 1991)*

### REFERENCES

Andreu-Sanchez J.L., Faro J., Alonso J.M., Paige C.J., Martinez-A. C., and Marcos M.A.R. (1990). Ontogenic characterization of thymic B lymphocytes. Analysis in different mouse strains. Eur. J. Immunol. **20:** 1767–1773.

Clarke A.G., and Kendall M.D. (1989). Histological changes in the thymus during mouse pregnancy. Thymus **14:** 65–78.

Compton M.M., Caron L.M., et al., (1987). Glucocorticoid action of the immune system. J. Steroid Biochem. **27:** 201–208.

Crispe I.N., Moore M.W., Husmann L.A., Smith L., Bevan M.J., and Shimonkevitz R.P. (1987). Differentiation potential of subsets of $CD4^-8^-$ thymocytes. Nature **329:** 336–339.

Dargemont C., Dunon D., Deugnier M.A., Denoyelle M., Girault J.M., Lederer F., Le K.H.D., Godeau F., Thiery J.P., and Imhof B.A. (1989). Thymotaxin, a chemotactic protein, is identical to beta-2 microglobulin. Science **246:** 803–805.

Deugnier M.A., Imhof B.A., Bauvois B., Dunon D., Denoyelle M., and Thiery J.P. (1989). Characterization of rat T cell precursors sorted by chemotactic migration toward thymotaxin. Cell **56:** 1073–1083.

Fowlkes B.J., Edison L., Mathieson B.J., and Chused T.M. (1985). Early T thymocytes: differentiation *in vivo* of adult intrathymic precursor cells. J. Exp. Med. **162:** 802–822.

Fowlkes B.J., and Pardoll D.M. (1989). Molecular and cellular events of T cell development. Adv. Immunol. **44:** 207–264.

Goldschneider I., Komschlies K., and Greiner, D.L. (1986). Studies of thymocytopoiesis in rats and mice. I. Kinetics of appearance of thymocytes using a direct intrathymic (i.t.) adoptive transfer assay for thymocyte precursors. J. Exp. Med. **163:** 1–17.

Green M.C. and Shultz L.D. (1975). Motheaten, an immunodeficient mutant of the mouse. I. Genetics and pathology. J. Hered. **66:** 250–258.

Greiner D.L., Goldschneider I., and Barton, R.W. (1982). Identification of thymocyte progenitors in hematopoietic tissues of the rat. II. Enrichment of functional prothymocytes on the fluorescence-activated cell sorter. J. Exp. Med. **156:** 1448–1460.

Greiner D.L., Goldschneider I., Komschlies K.L., Medlock E.S., Bollum F.J., and Shultz L.D. (1986). Defective lymphopoiesis in bone marrow of motheaten (*me/me*) and viable motheaten (*me^v/me^v*) mutant mice. I. Analysis of development of prothymocytes, early B lineage cells, and terminal deoxynucleotidyl transferase-positive cells. J. Exp. Med. **164:** 1129–1144.

Harr J.L., Popp J.D., and Shultz L.D. (1989). Defective in vitro migratory capacity of bone marrow cells from viable motheaten mice in response to normal thymus culture supernatants. Exp. Hematol. **17:** 21–24.

Hayashi S.I., Witte P.L., Shultz L.D., and Kincade P.W. (1988). Lymphopoiesis in culture is prevented by interaction with adherent bone marrow cells from mutant viable motheaten mice. J. Immunol. **140:** 2139–2147.

Hirokawa K., Kubo S., Utsuyama M., Kurashima C., and Sado T. (1986). Age-related change in the potential of bone marrow cells to repopulate the thymus and splenic T cells in mice. Cell. Immunol. **100:** 443–451.

Imhof B.A., Deugnier M.A., Girault J.M., Champion S., Damais C., Itoh T., and Thiery J.P. (1988). Thymotaxin: A thymic epithelial peptide chemotactic for T-cell precursors. Proc. Natl. Acad. Sci. USA **85:** 7699–7703.

Kadish J.L., and Basch R.S. (1977). Hematopoietic thymocyte precursors. III. A population of thymocytes with the capacity of return ("home") to the thymus. Cell. Immunol. **30:** 12–24.

Kay M.M.B. (1984). Immunological aspects of aging: Early changes in thymic activity. Mech. Aging Develop. **28:** 193–218.

Kincade P.W. (1981). Hematopoietic abnormalities in New Zealand Black and motheaten mice. *In:* Immunological Defects in Laboratory Animals, Gershwin M.E., and Merchant B. Eds. (New York: Plenum Press), pp. 125–142.

Kincade P.W. (1987). Experimental models for understanding B lymphocyte formation. Adv. Immunol. **41:** 181–267.

Koller B.H., Marrack P., Kappler J.W., and Smithies O. (1990). Normal development of mice deficient in beta-2 M, MHC class I proteins, and $CD8^+$ T cells. Science **248:** 1227–1230.

Komschlies K.L., Greiner, D.L., Shultz L.D., and Goldschneider I. (1987). Defective lymphopoiesis in the bone marrow of motheaten (*me/me*) and viable motheaten (*me^v/me^v*) mutant mice. III. Normal mouse bone marrow cells enable *me^v/me^v* prothymocytes to generate thymocytes after intravenous transfer. J. Exp. Med. **166:** 1162–1167.

Landreth K.S., McCoy K., Clagett J., Bollum F.J., and Rosse C. (1981). Deficiency in cells expressing terminal transferase in autoimmune (motheaten) mice. Nature (London). **290:** 409–411.

McCoy K.L., Chi E., Engel D., Rosse C., and Claget J. (1982). Abnormal *in vitro* proliferation of splenic mononuclear phagocytes from autoimmune motheaten mice. J. Immunol. **128:** 1797–1804.

McCoy K.L., Clagett J., and Rosse C. (1985). Effects of the motheaten gene on murine B-cell production. Exp. Hematol. **13:** 554–559.

McCoy K.L., Nielson K., and Clagett J. (1984). Spontaneous production of colony-stimulating activity by splenic MAC-1 antigen-positive cells from autoimmune motheaten mice. J. Immunol. **132:** 272–276.

Medlock E.S., Goldschneider I., Greiner D.L., and Shultz L.D. (1986). Defective microenvironmental control of lymphopoiesis in aged and motheaten mice. *In:* Sixth International Congress of Immunology (New York: Academic Press), pp. 1.62.36.

Medlock E.S., Goldschneider I., Greiner D.L., and Shultz L.D. (1987). Defective lymphopoiesis in the bone marrow of motheaten (*me/me*) and viable motheaten (*me^v/me^v*) mutant mice. II. Description of a microenvironmental defect for the generation of terminal deoxynucleotidyl transferase-positive bone marrow cells in vitro. J. Immunol. **138:** 3590–3597.

Morse H.C., Shen F.-W., and Hammerling U. (1987). Genetic nomenclature for loci controlling mouse lymphocyte antigens. Immunogenetics **25:** 71–78.

Nagelkerken L., Hertogh-Huijbregts A., Dobber R., and Drager A. (1991). Age related changes in lymphokine production related to a decreased number of $CD45RB^{hi}$ $CD4^+$ T cells. Eur. J. Immunol. In press.

Reichert R.A., Weissman I.L., and Butcher E.C. (1986). Dual immunofluorescence studies of cortisone-induced thymic involution: Evidence for a major cortical component to cortisone-resistant thymocytes. J. Immunol. **136:** 3529–3534.

Robey E.A., Fowlkes B.J., and Pardoll D.M. (1990). Molecular mechanisms for lineage commitment in T cell development. Sem. Immunol. **2:** 25–34.

Santisteban G.A. (1960). The growth and involution of lymphatic tissue and interrelationships to aging and to the growth of the adrenal glands and sex organs in CBA mice. Anat. Rec. **136:** 117–123.

Scollay R., and Shortman K. (1984). Cell traffic in the adult thymus: Cell entry and exit, cell birth and death. *In:* Recognition and Regulation in Cell Mediated Immunity, Watson J.D., and Marbrook J., Eds. (New York: Marcel Dekker), pp. 3–30.

Scollay R., and Shortman K. (1985). Identification of early stages of T lymphocyte development in the thymic cortex and medulla. J. Immunol. **134:** 3632–3642.

Scollay R., Smith J., and Stauffer V. (1986). Dynamics of early T cells: Prothymocyte migration and proliferation in the adult mouse thymus. Immunol. Rev. **91:** 129–157.

Scollay R., Wilson A., D'Amico A., Kelly K., Egerton M., Pearse M., Wu L., and Shortman K. (1988). Developmental status and reconstitution potential of subpopulations of murine thymocytes. Immunol. Rev. **104:** 81–120.

Shortman K., Egerton M., Spangrude G.J., and Scollay R. (1990). The generation and fate of thymocytes. Sem. Immunol. **2:** 3–12.

Shortman K., Wilson A., Pearse M., Gallagher P., and Scollay R. (1988). Mouse strain differences in subset distribution and T cell antigen receptor expression among $CD4^-CD8^-$ thymocytes. Immunol. Cell. Biol. **66:** 423–433.

Shultz L.D. (1988). Pleiotropic effects of deleterious alleles at the "motheaten" locus. Curr. Top. Microbiol. Immunol. **137:** 216–222.

Shultz L.D., Coman D.R., et al. (1984). "Viable motheaten," a new allele at the motheaten locus. I. Pathology. Am. J. Pathol. **116:** 179–192.

Shultz L.D., and Sidman C.L. (1987). Genetically determined murine models of immunodeficiency. Annu. Rev. Immunol. **5:** 367–403.

Spangrude G.J., and Scollay R. (1990). Differentiation of hematopoietic stem cells in irradiated mouse thymic lobes. Kinetics and phenotype of progeny. J. Immunol. **145:** 3661–3668.

Tyan M.L. (1982). Age-related changes in *in vitro* and *in vivo* survival of murine CFU-S and CFU-c. Mech. Ageing Dev. **19:** 279–287.

Van Vliet E., Melis M., and van Ewijk W. (1986). The influence of dexamethasone treatment on the lymphoid and stromal composition of the mouse thymus: A flow cytometric and immunohistological analysis. Cell. Immunol. **103:** 229–240.

Van Zant G., and Shultz L.D. (1989). Hematologic abnormalities of the immunodeficient mouse mutant, viable motheaten (*me^v*). Exp. Hematol. **17:** 81–87.

Von Boehmer H. (1990). Developmental biology of T cells in T cell receptor transgenic mice. Ann. Rev. Immunol. **8:** 531–556.

Williams L.H., Udupa K.B., and Lipschitz D.A. (1986). Evaluation of the effect of age on hematopoiesis in the C57B1/6 mouse. Exp. Hematol. **14:** 827–832.

Wilson A., Ewing T., Owens T., Scollay R., and Shortman K. (1988). Subpopulation of early thymocytes. A cross-correlation flow cytometric analysis of adult mouse Ly-2$^-$ L3T4$^-$ (CD8$^-$CD4$^-$) thymocytes using eight different surface markers. J. Immunol. **140:** 1461–1469.

Zijlstra M., Box M., Simister N.E., Loring J.M., Raulet D.H., and Jaenisch R. (1990). Beta-2 microglobulin deficient mice lack CD4$^-$CD8$^+$ cytolytic T cells. Nature **344:** 742–746.

